# Image-guided selection of Gd@C-dots as sensitizers to improve radiotherapy of non-small cell lung cancer

**DOI:** 10.1186/s12951-021-01018-9

**Published:** 2021-09-22

**Authors:** Xiaofen Ma, Chaebin Lee, Tao Zhang, Jinghua Cai, Hui Wang, Fangchao Jiang, Zhanhong Wu, Jin Xie, Guihua Jiang, Zibo Li

**Affiliations:** 1 Department of Nuclear Medicine, Guangdong Second Provincial General Hospital, 466 Xingang Middle Road, Haizhu District, Guangdong Province 510317 Guangzhou City, People’s Republic of China; 2 Department of Radiology, Biomedical Research Imaging Center, and Lineberger Comprehensive Cancer Center, University of North Carolina at Chapel Hill, 125 Mason Farm Road, Chapel Hill, NC 27599 USA; 3 Department of Chemistry, University of Georgia, 140 Cedar Street, Athens, GA 30602 USA

**Keywords:** Gadolinium, Carbon dots, Radiosensitizers, PET imaging, Non-small cell lung cancer, Radiation therapy

## Abstract

**Background:**

Recently, gadolinium-intercalated carbon dots (Gd@C-dots) have demonstrated potential advantages over traditional high-Z nanoparticles (HZNPs) as radiosensitizers due to their high stability, minimal metal leakage, and remarkable efficacy.

**Results:**

In this work, two Gd@C-dots formulations were fabricated which bore carboxylic acid (CA-Gd@C-dots) or amino group (pPD-Gd@C-dots), respectively, on the carbon shell. While it is critical to develop innovative nanomateirals for cancer therapy, determining their tumor accumulation and retention is equally important. Therefore, *in vivo* positron emission tomography (PET) was performed, which found that ^64^Cu-labeled pPD-Gd@C-dots demonstrated significantly improved tumor retention (up to 48 h post injection) compared with CA-Gd@C-dots. Indeed, cell uptake of ^64^Cu-pPD-Gd@C-dots reached close to 60% of total dose compared with ~ 5% of ^64^Cu-CA-Gd@C-dots. pPD-Gd@C-dots was therefore further evaluated as a new radiosensitizer for non-small cell lung cancer treatment. While single dose radiation plus intratumorally injected pPD-Gd@C-dots did lead to improved tumor suppression, the inhibition effect was further improved with two doses of radiation. The persistent retention of pPD-Gd@C-dots in tumor region eliminates the need of reinjecting radiosensitizer for the second radiation.

**Conclusions:**

PET offers a simple and straightforward way to study nanoparticle retention *in vivo*, and the selected pPD-Gd@C-dots hold great potential as an effective radiosensitizer.

**Graphic abstract:**

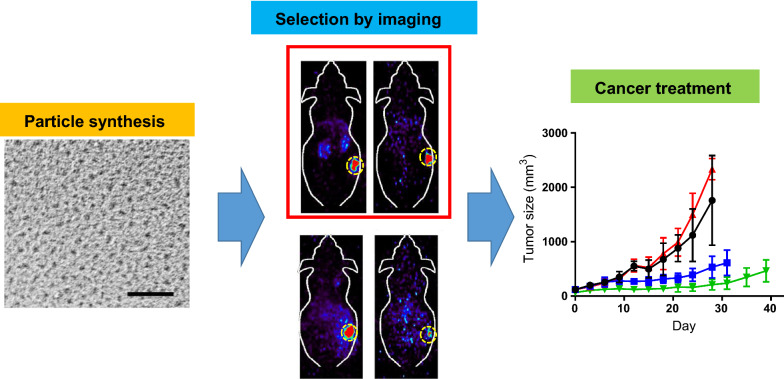

**Supplementary Information:**

The online version contains supplementary material available at 10.1186/s12951-021-01018-9.

## Introduction

Radiation therapy (RT) is one of the most widely used cancer treatment modalities [1–3]. However, significant side effects are often observed due to RT-induced damage to surrounding normal tissues [4–7]. Selectively increasing energy deposition at the tumor region could potentially improve tumor treatment efficacy and reduce the associated side effects. Over the last decade, various nanomedicines have been developed and tested as radiosensitizing agents, among which high-Z nanoparticles (HZNPs) have attracted extensive attention [8–13]. HZNPs afford large cross-section for high energy photons, thus producing more photo- or Auger-electrons (the effect of which is varied by Z^3^ or Z^4^) under radiation that damage cancer cells. Such radiosensitizing effects were observed in HZNPs made of gold, silver, bismuth, and gadolinium [14–19]. Excitingly, NBTXR3, a hafnium oxide formulation, has demonstrated clinical benefits and received regulatory approval in the EU for enhancing RT [20, 21].

Despite promising results, many conventional HZNPs are associated with issues such as a large particle size, uncontrolled metal leakage, and heavy-metal toxicity [22–25]. Moreover, dose modifying factors of nanoparticles are hard to predict without a costly and lengthy animal experiments [8, 26, 27]. In this report, we tested two ultrasmall Gd-encapsulated carbon dots (Gd@C-dots), CA-Gd@C-dots and pPD-Gd@C-dots, which bear positive and neutral surface charges, respectively. We speculated that in addition to particle size, surface properties could impact the *in vitro* and *in vivo* behaviors of Gd@Cdots and in turn affect their role as a radiosensitizer. Positron emission tomography (PET) is a noninvasive imaging technology that allows us to track radiolabeled ligands and materials quantitatively and repetitively. In this report, we labeled both CA-Gd@C-dots and pPD-Gd@C-dots with ^64^Cu and investigated the nanoparticles’ uptake by cancer cells and retention in tumors using PET. We exploit this quantitative molecular imaging method to monitor nanoparticle clearance after injection and screen for a better formulation. Overall, the research not only evaluated new radiosensitizers based on Gd@C-dots, but also demonstrated that PET could be used as a tool to examine clearance of intratumorally injected nanoparticles so as to facilitate nanomedicine research.

## Materials and methods

### Materials

All chemicals and solvents were obtained from commercial sources and used without further purification. p-Phenylenediamine (pPD, Sigma Aldrich, Cat# 78429), Citric acid (CA, Sigma Aldrich, Cat# 251275), Gadolinium nitrate hexahydrate (Gd(NO_3_)_3_∙6H_2_O, Sigma Aldrich, Cat# 211591), Ethanol (KOPTEC, Cat# 19J14D), Dialysis membrane (Spectrum, [MWCO] = 100–500), Milli-Q H_2_O, 3-(4,5-Dimethylthiazolyl-2)-2,5-diphenyltetrazolium bromide (MTT) (Sigma Aldrich, Cat# M2128).

All experimental procedures involving animals were executed accordance with animal protocol approved by University of North Carolina at Chapel Hill Institutional Animal Care and Use Committee.

### The synthesis of CA-Gd@C-dots and pPD-Gd@C-dots

Gd@C-dots were synthesized by hydrothermal reaction. Briefly, 1.33 mmol of Gd(NO_3_)_3_∙6H_2_O and 1.48 mmol of carbon precursor (pPD or CA) was dissolved in 60 mL of either ethanol or Milli-Q H_2_O by vortex mixer, and the solution was transferred into a 100 mL poly(tetrafluoroethylene)-lined autoclave. The autoclave was kept in an oven, and the temperature was increased at a rate of 3 °C/min to 180 °C. After 12 h, the autoclave was cooled down to room temperature. The raw products were washed three times with water, followed by dialysis (MWCO 500) that removed carbon byproducts and free gadolinium ions. The final products were lyophilized and stored at − 80 °C.

### Characterization of CA-Gd@C-dots and pPD-Gd@C-dots

Transmission electron microscopy (TEM) images were acquired on a FEI TECNAI 20 transmission electron microscope operated at 200 kV. The zeta potential of the nanoparticles was measured by a Malvern Zetasizer Nano ZS system. Energy-dispersive X-ray spectroscopy (EDS) and element mapping were performed on a FEI Inspect F FEG-SEM system equipped with EDZX EDS. Inductively coupled plasma mass spectrometry (ICP-MS) was used to quantify Gd content in the samples.

### Preparation of DOTA conjugated Gd@C-dots

DOTA-CA-Gd@C-dots was prepared according to the reference procedure [28]. Briefly, CA-Gd@C-dots was activated by EDC and sNHS at pH 5.5 for 30 min with a CA-Gd@C-dots:EDC:sNHS molar ratio of 10:5:4. The activated CA-NHS-Gd@C-dots) (0.92 µmol, calculated on the basis of sNHS, 300 µL) were then added into a 200 µL DOTA-Amine (MW 766.4, 500 µg, 0.65 µmol) solution at a pH of 8.5. The mixture was incubated at 4 °C for 12 h, and the unreacted materials were removed using a 3 k, 4 mL micro filter unit (dilute the reaction solution to 4 mL by adding deionized water; centrifuge at 3500 rpm for 15 min; and disperse using pipette. Repeat this procedure three times until 250 µL of particle was left on the filter).

DOTA-NHS (100 µg, 0.13 µmol) was added into 200 µL pPD-Gd@C-dots NPs (2.2 µmol, calculated on the basis of –NH_2_ group) solution at a pH of 8.5. The mixture was incubated at 4 °C for 12 h, and the unreacted materials were removed using a 10 k, 4 mL micro filter unit (dilute the reaction solution to 4 mL by adding deionized water; centrifuge at 3500 rpm for 15 min and disperse using pipette. Repeat this three times until 100 µL of particle was left on the filter) to obtain DOTA-pPD-Gd@C-dots. Similarly, NOTA-NCS (50 µg, 0.09 µmol) was added into 200 µL pPD-Gd@C-dots (2.2 µmol, calculated on the basis of –NH_2_ group) solution at a pH of 9.5. The mixture was incubated at 4 °C for 12 h, and the unreacted materials were removed using a 10 k, 4 mL micro filter unit (dilute the reaction solution to 4 mL by adding deionized water; centrifuge at 3500 rpm for 15 min and disperse using pipette. Repeat this three times until 100 µL of particle was left on the filter) to obtain NOTA-pPD-Gd@C-dots.

### Radiochemistry

^64^Cu-DOTA-CA-Gd@C-dots, ^64^Cu-DOTA-pPD-Gd@C-dots and ^64^Cu-NOTA-pPD–Gd@C-dots (50 µL, respectively) were prepared by the addition of ^64^Cu (2 mCi, respectively) and chelator modified particles in 0.25 M ammonium acetate (pH 5.5) buffer, and the mixture was incubated for 30 min at 40 °C. ^64^Cu labeled Gd@C-dots were then purified using a micro filter unit with deionized water as the washing solution. The radioactive fractions containing ^64^Cu-DOTA-Gd@C-dots were collected for further *in vitro* and *in vivo* experiments.

### Cells and animals

H1299 is a human NSCLC cell line. H1299 cells were grown in RPMI 1640 medium supplemented with 10% FBS and maintained in a humidified, 5% carbon dioxide (CO_2_) atmosphere at 37 °C until sufficient cells were available.

To generate H1299 tumor model, the female nude mice were injected subcutaneously with H1299 cells at a concentration of 2 × 10^6^ cells in 100 μL of phosphate buffered saline in the thigh. Tumors were established within 3–4 week after inoculation (the tumor volume, about 100 mm^3^). The mice were subjected to small-animal PET and therapy studies.

### In vitro toxicity study

The cell viability was studied with H1299 cells line following to the standard MTT assays. The H1299 cells were seeded on clear 96-well plates (8000 cells per well) in 100 µL cell culture media. After 24 h, 100 µL of Gd@C-dots (either CA or pPD) containing cell culture media in range of concentration (0–400 µg/mL, Gd) were added to each well and incubated at 37 °C with the cells for 24 h. Then, 10 µL of MTT solution (10 mg/mL) was added to each well and stored in the incubator for another 4 h. All solution was aspirated, and 100 µL of DMSO was added to each well. The plate was shaked for 10 min, and the absorbance at 570 nm was read by BioTek Synergy MX multi-mode microplate reader.

### ATP assay for cytotoxicity evaluation with H1299 cells

The cell viability was evaluated on H1299 cells using standard 1Step ATP assay (PerkinElmer, ATPlite 1step Luminescence Assay Cat#6016736). The H1299 cells were seeded on opaque 96-well plates (8000 cells per well) in 100 µL cell culture media. After 24 h, 100 µL of Gd@C-dots (either CA or pPD) at a concentrations (0–400 µg/mL, Gd) were added into each well and incubated at 37 °C with the cells for 24 h. Then, cell medium were removed and cells were rinsed with PBS three times before 100 µL of ATP assay buffer was added to each well (Additional file 1: Fig S2). The plate was kept in dark for 10 min and the overall luminescence was read by a BioTek Synergy MX multi-mode microplate reader.

### Cell uptake

For cell uptake assay, H1299 cells were seeded into a 24-well plate at a density of 1 × 10^5^ cells per well and incubated with ~ 3 µCi ^64^Cu-DOTA-CA-Gd@C-dots, ^64^Cu-DOTA-pPD-Gd@C-dots and ^64^Cu-NOTA-pPD-Gd@C-dots at 37 °C. After incubation for 5, 15, 30, 60, 120, and 240 min, the tumor cells were washed twice with cold PBS and harvested by adding 250 µL 2 N NaOH. The total cell lysate at each time point was collected and the radioactivity was measured in a gamma counter. The cell uptake was expressed as the percentage of the added dose after decay correction. Each data point is an average of quadruplicates.

### In vivo small-animal PET studies

PET scans and image analysis were performed as previously reported [29, 30]. For small-animal PET imaging, H1299 tumor model mice were intratumorally injected with 2.15 MBq of ^64^Cu-DOTA-CA-Gd@C-dots and 0.89 MBq ^64^Cu-DOTA-pPD-Gd@C-dots. Serial imaging (1, 4, 24 and 48 h after injection; static scan duration 10, 10, 10, and 30 min, respectively) was performed using a small-animal PET scanner (Super Argus). Next, the images were reconstructed using a 2-dimensional ordered-subsets expectation maximization algorithm. Next, for each image, regions of interest (ROIs) were drawn over the tumor, major tissue, and organ using amide software. Assuming a tissue density of 1 g/mL, the means were converted to counts per gram per minute. Dividing counts per gram per minute by injected dose gave the image ROI-derived percentage injected dose per gram tissue (%ID/g) values.

### X-ray radiotherapy studies

H1299 subcutaneous mouse model was used for the therapy study. Twelve mice with tumors of approximately 100 mm^3^ were randomly divided into four groups. For the treatment group, the mice were first intratumorally injected with 100 µg pPD-Gd@C-dots (4 mg/mL, 25 μL). After 1 h, X-ray irradiation (6 Gy) was applied to tumors, with the rest of the animal body covered by 6-layer lead. The three control groups received: (1) control; (2) 100 µg pPD-Gd@C-dots alone; (3) X-Ray irradiation (6 Gy) only. After the treatment, to monitor tumor growth, the tumor sizes and body weights were measured at different time points. Tumor sizes were measured by a caliper and calculated using this formula: size (mm^3^) = length (mm) × width (mm)^2^/2.

In addition, we used 12 H1299 mice for another treatment study. For the treatment group, the mice were first intratumorally injected with 100 μg pPD-Gd@C-dots (4 mg/mL, 25 μL). After 1 h, X-Ray irradiation (6 Gy) was applied to tumors, with the rest of the animal body covered by 6-layer lead. Three days later, the same treatment was given to these mice. The three control groups received: (1) Control; (2) 100 µg pPD-Gd@C-dots alone; (3) X-ray irradiation (6 Gy) only (twice). After the treatment, the tumor sizes and body weights were measured at different time points in the same way.

## Results and discussion

### Preparation and characterizations of Gd@C-dots

Gd@C-dots were synthesized through hydrothermal reaction using p-phenylenediamine or citric acid as carbon precursor and Gd(NO_3_)_3_ as gadolinium precursor [31–33]. After reaction, the raw products were purified by dialysis to remove unreacted precursors and surface-bound metals. Both types of nanoparticles can be stably dispersed in water. While pPD-Gd@C-dots were red in color, CA-Gd@C-dots appeared brown (Additional file 1: Fig. S1).

**Fig. 1:**
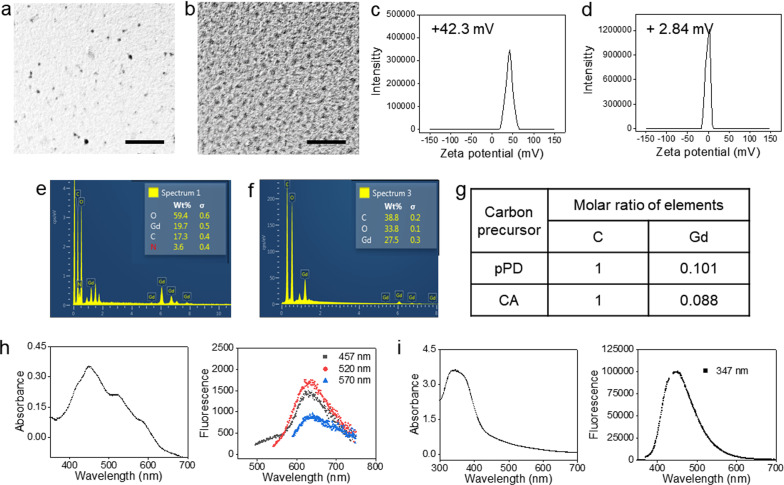
Synthesis and characterizations of Gd@C-dots. **a**, **b** Representative TEM images of pPD-Gd@C-dots (**a**) and CA-Gd@C-dots (**b**). The scale bars are 100 nm. **c**, **d** DLS analysis. pPD-Gd@C-dots (**c**) and CA-Gd@C-dots (**d**) bear positive (+ 42.3 mV) and almost neutral (+ 2.84) surfaces, respectively. **e**, **f** EDS elemental analysis of pPD-Gd@C-dots (**e**) and CA-Gd@C-dots (**f**). **g** Table showing compositions of pPD-Gd@C-dots and CA-Gd@C-dots, based on EDS results. **h** Uv–vis absorption (left) and fluorescence (right) spectra of pPD-Gd@C-dots. pPD-Gd@C-dots have absorbance peak at 457 nm, 520 nm, and 570 nm, and an emission peak at ~ 630 nm (excited at 457, 520, or 570 nm). (i) Uv–vis absorption (left) and fluorescence (right) spectra of CA-Gd@C-dots. CA-Gd@C-dots have an absorbance peak at 347 nm and emission peak at 449 nm (excited at 347 nm)

Transmission electron microscopy (TEM) found that both nanoparticles have a diameter of ~ 3 nm (Fig. 1a, b). Zeta potential analysis found that pPD-Gd@C-dots are positively charged on the surface (+ 42.3 mV, Fig. 1c), while CA-Gd@C-dots are virtually neutral (+ 2.84 mV, Fig. 1d). The positive surface charge of the former is attributed to surface amine groups inherited from the pPD precursor. Energy dispersive spectroscopy (EDS) found that the C-to-Gd molar ratio was similar, which is 1:0.1 for pPD-Gd@C-dots and 1:0.09 for CA-Gd@C-dots (Fig. 1e–g).

The optical properties are also different between the two nanoparticles. Specifically, pPD-Gd@C-dots showed an absorbance peak at 457 nm, and two shoulders at 520 and 570 nm, respectively (Fig. 1h). Meanwhile, an emission peak at ~ 630 nm was observed. For Gd@C-dots made with CA, on the other hand, the absorbance peak appears at 347 nm, and the emission peak is centered at 449 nm (Fig. 1i).

These results indicate that two ultrasmall Gd@C-dots were successfully synthesized and they showed varied surface and optimal properties.

### Chemistry and radiochemistry

Before *in vivo* therapy experiment, we aim to evaluate the cell uptake and clearance profile of the Gd@C-dots using radiolabeling approach. Therefore, radiometal chelators were introduced to the nanoparticles through amide bond formation using reported procedures [34–36]. In brief, the carboxylic acid group in CA-Gd@C-dots was activated by EDC which was then conjugated with DOTA-NH_2_, leading to DOTA-CA-Gd@C-dots. Similarly, the amino group in pPD-Gd@C-dots reacted with DOTA-sNHS ester or NOTA-NCS leading to DOTA-pPD-Gd@C-dots or NOTA-pPD-Gd@C-dots ready for ^64^Cu labeling. The chelator modified nanomaterials could all be efficiently labeled by ^64^Cu with a yield ranging from 75%-90% (non-decay corrected).

### In vitro experiments

#### Cells and animals

**Fig. 2:**
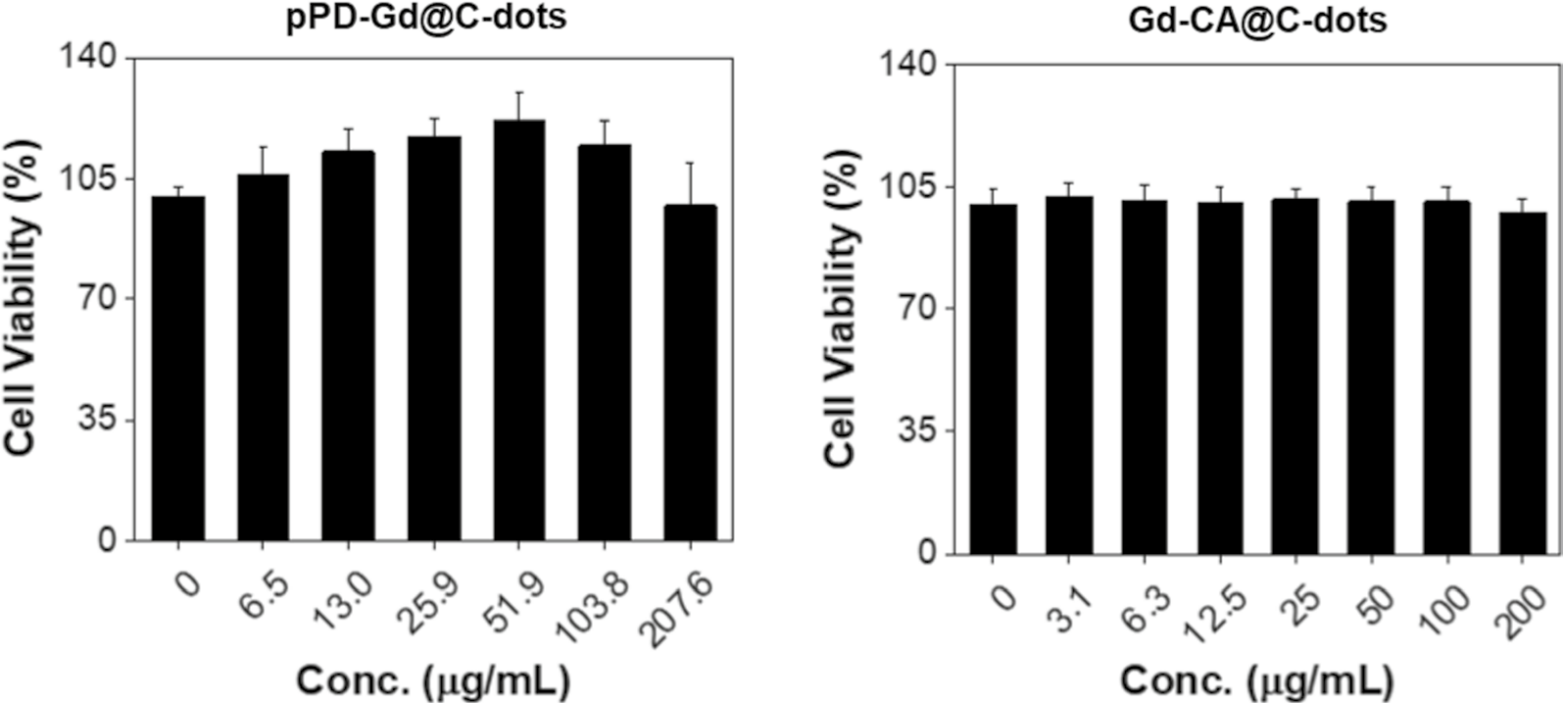
Cytotoxicity of pPD-Gd@C-dots and CA-Gd@C-dots, measured by MTT assay at 24 h on H1299 cells. No significant toxicity was observed up to 200 µg Gd/mL

**Fig. 3:**
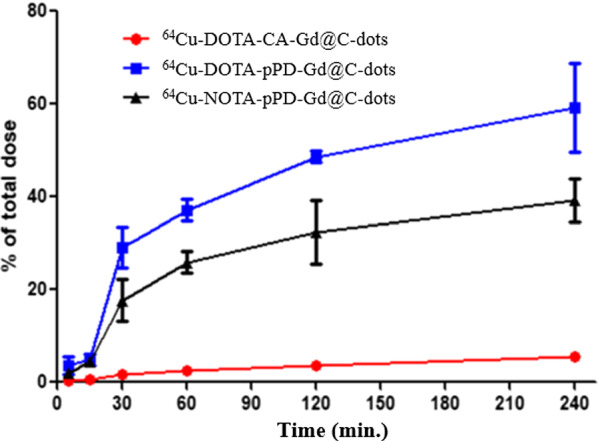
Cell uptake assay of ^64^Cu labeled Gd@C-dots in H1299 cells

We investigated safety and efficacy of pPD-Gd@C-dots and CA-Gd@C-dots with H1299 cells, which is a human non-small cell lung cancer cell line. Both nanoparticles were non-toxic to H1299 cells (Fig. 2; Additional file 1: S2). To investigate the cell uptake difference between CA-Gd@C-dots and pPD-Gd@C-dots, we first incubated the ^64^Cu labeled agents with H1299 cells. As shown in Fig. 3, ^64^Cu-DOTA-CA-Gd@C-dots had lowest cell uptake at all time points tested, with the highest uptake observed being 5.6 ± 0.2% of total activity at 240 min. The cell uptake of ^64^Cu-DOTA-pPD-Gd@C-dots was significantly higher than ^64^Cu-DOTA-CA-Gd@C-dots, which was 1.9 ± 1.3, 4.5 ± 1.3, 17.7 ± 9.1, 25.9 ± 4.6, 32.4 ± 13.6, and 39.2 ± 9.3% of total activity after 5, 15, 30, 60, 120, and 240 min incubation, respectively. The cell uptake of ^64^Cu-NOTA-pPD-Gd@C-dots was the highest among three tracers, which was 3.6 ± 3.9, 4.9 ± 2.4, 29.1 ± 8.8, 37.2 ± 4.5, 48.7 ± 2.6, and 59.2 ± 18.9, after 5, 15, 30, 60, 120, and 240 min incubation, respectively. In summary, ^64^Cu labeled pPD-Gd@C-dots demonstrated more than 10 times higher cell uptake *in vitro* than ^64^Cu labeled CA-Gd@C-dots. It is still unknown why ^64^Cu-DOTA-CA-Gd@C-dots showed higher uptake than ^64^Cu-NOTA-CA-Gd@C-dots. Because only small percent of surface amino groups were modified, it is unlikely lead to significant changes on surface charges. The observed cell uptake difference may be attributed to the stability difference between Cu-DOTA and Cu-NOTA complex, which still need to be confirmed in future studies. Because we did not observe significant difference between DOTA and NOTA modified pPD-Gd@C-dots, ^64^Cu-DOTA-pPD-Gd@C-dots was used for *in vivo *imaging studies to minimize potential impact from the chelator and made it comparable with ^64^Cu-DOTA-CA-Gd@C-dots.

### In vivo small-animal PET studies

**Fig. 4:**
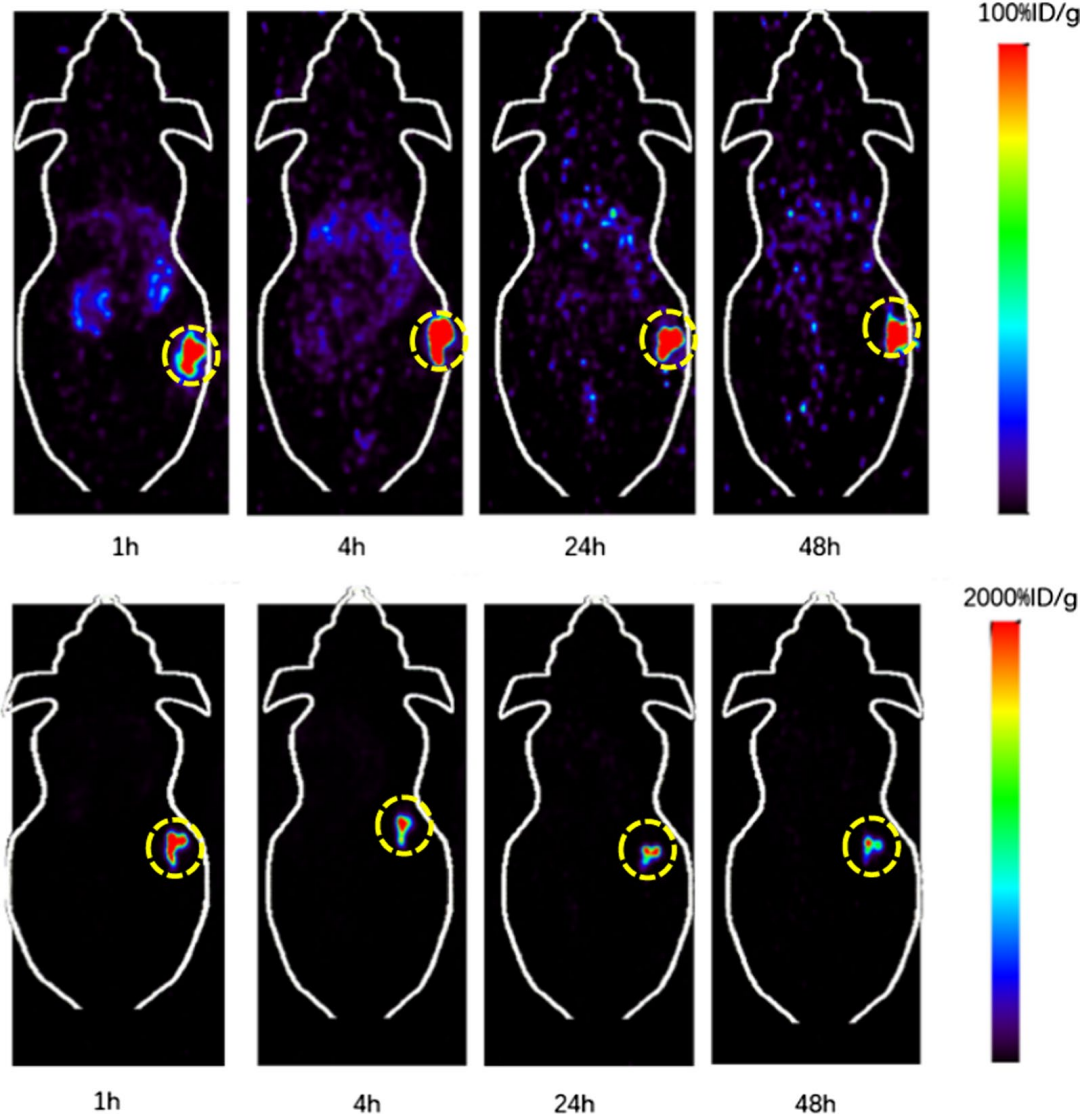
Representative coronal small-animal PET images of mice bearing H1299 xenografts at 1, 4, 24, and 48 h after Intratumoral injection of 0.89 MBq of ^64^Cu-DOTA-pPD-Gd@C-dots with scope bar 100%ID/g(top) and 2000%ID/g (bottom)

**Fig. 5:**
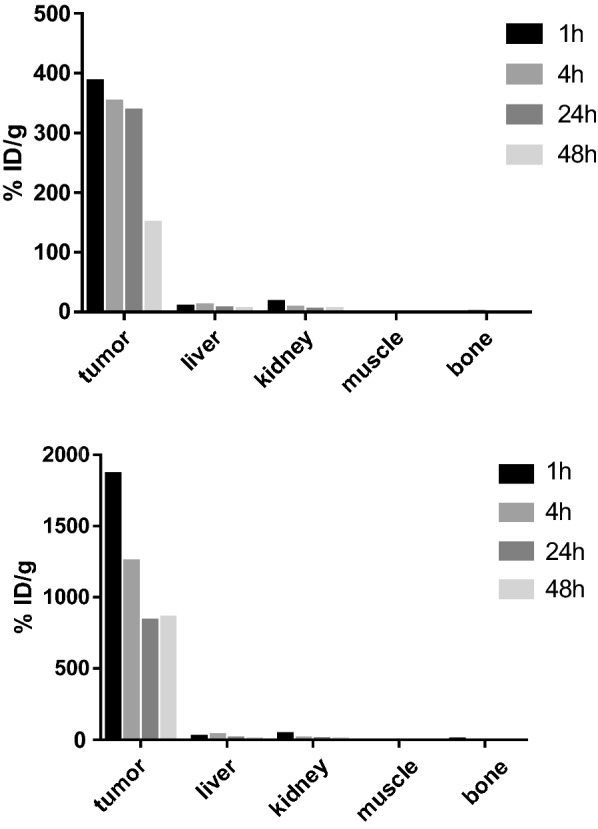
Comparison of tumor, liver, kidney, muscle and bone uptake of ^64^Cu-DOTA-pPD-Gd@C-dots at different time points (1, 4, 24, and 48 h) after intratumoral injection

To evaluate whether the increased cell trapping/retention could also lead to significantly increased tumor retention in vivo, we studied the distribution of ^64^Cu-DOTA-CA-Gd@C-dots and ^64^Cu-DOTA-pPD-Gd@C-dots using small-animal PET in mice bearing H1299 xenografts at 1, 4, 24, and 48 h after Intratumoral injection. As shown in Figs. 4, 5, the tumor uptake of ^64^Cu-DOTA-pPD-Gd@C-dots showed minimal clearance within 24 h after injection (386.4, 352.9, and 337.4%ID/g at 1, 4, 24 h p.i. respectively) which maintained at 149.3%ID/g at 48 h p.i.. Compared to tumor, the uptakes in kidneys, liver, muscle and bone were almost negligible (Figs. 4, 5).

**Fig. 6:**
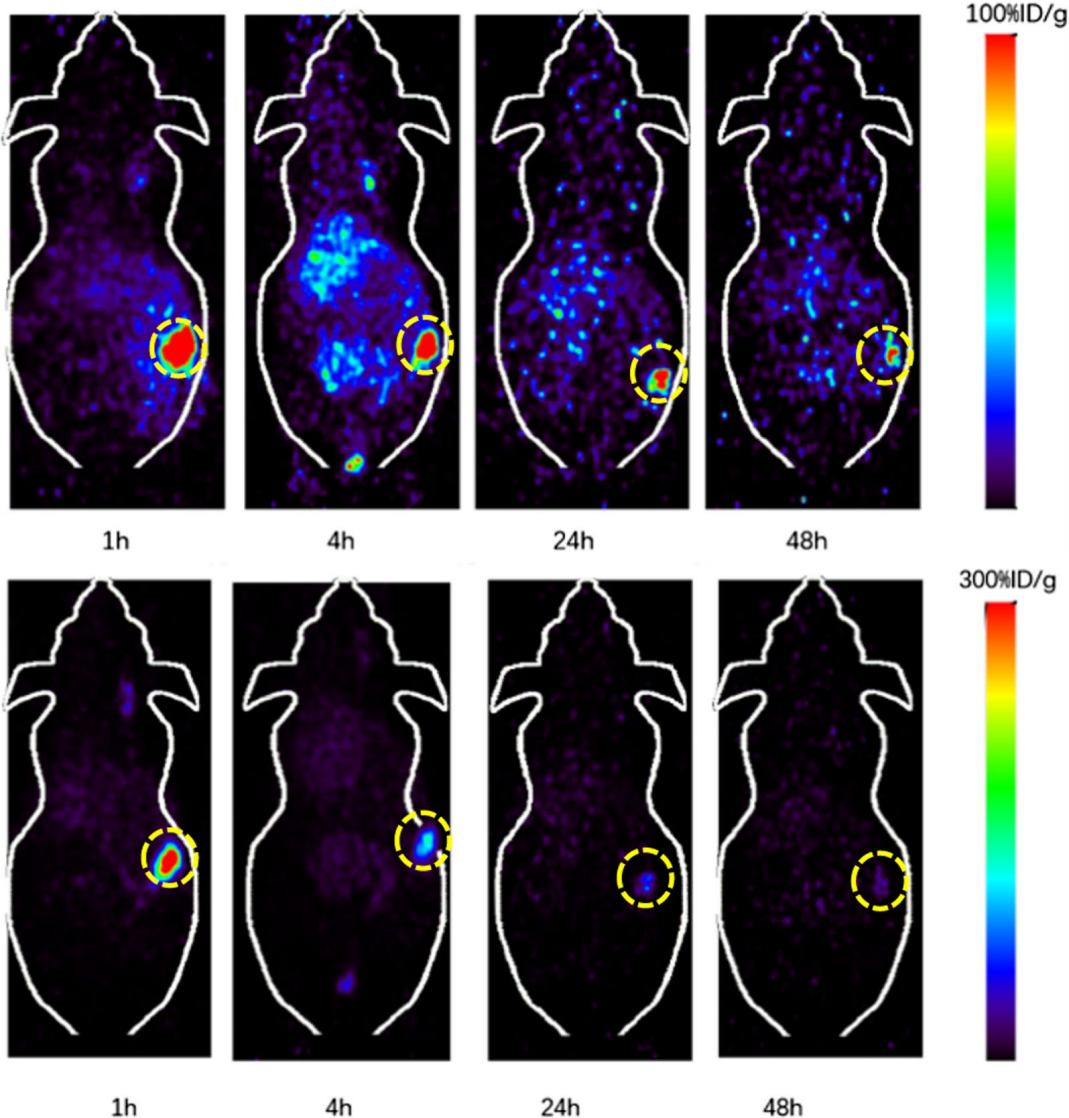
Representative coronal small-animal PET images of mice bearing H1299 xenografts at 1, 4, 24, and 48 h after intratumoral injection of 0.89 MBq of ^64^Cu-DOTA-CA-Gd@C-dots with scope bar100%ID/g(top) and 300%ID/g (bottom)

**Fig. 7:**
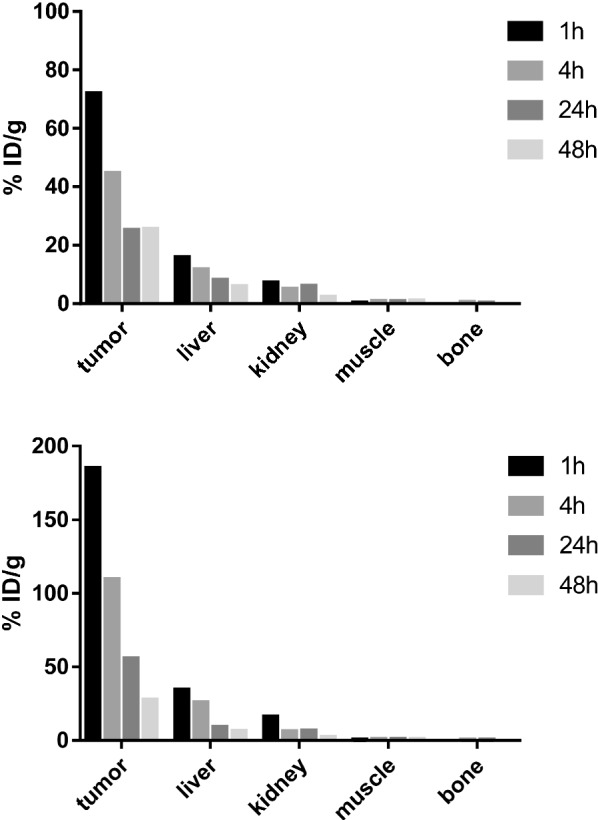
Comparison of tumor, liver, kidney, muscle and bone uptake of ^64^Cu-DOTA-CA-Gd@C-dots at different time points (1, 4, 24, and 48 h) after intratumoral injection

In comparison, ^64^Cu-DOTA-CA-Gd@C-dots demonstrated much faster clearance from the injection site. The tumor uptake in H1299 xenografts was 72.0, 44.7, 25.2 and 25.1%ID/g at 1, 4, 24, and 48 h after intratumoral injection, respectively (Fig. 6). Different from ^64^Cu-DOTA-pPD-Gd@C-dots, we did observe ^64^Cu-DOTA-CA-Gd@C-dots demonstrated apparent uptake in kidneys and liver through the blood circulation. The kidney, liver, muscle and bone uptake were 15.9, 7.2, 0.3, and 0.02%ID/g at 1 h after injection (Fig. 7). We like to point out that only one animal was used in each group, which showed large difference between two formulations. Although the obtained information is sufficient to guide us select the most promising formulation for therapy applications, additional numbers of animals would be needed if statistical analysis is required in future imaging analysis and comparison. In summary, ^64^Cu-DOTA-pPD-Gd@C-dots showed high and persistent tumor retention after injection, which makes it more suitable for *in vivo* treatment of tumors in following studies. Due to small particle size, both Figs. 4 and 6 shows that renal excretion is one of the main mechanisms for removing the radio drugs and nanoparticles from the body (especially after 4 h), This observation was also observed previously by other groups [37].

### X-ray radiotherapy studies

**Fig. 8:**
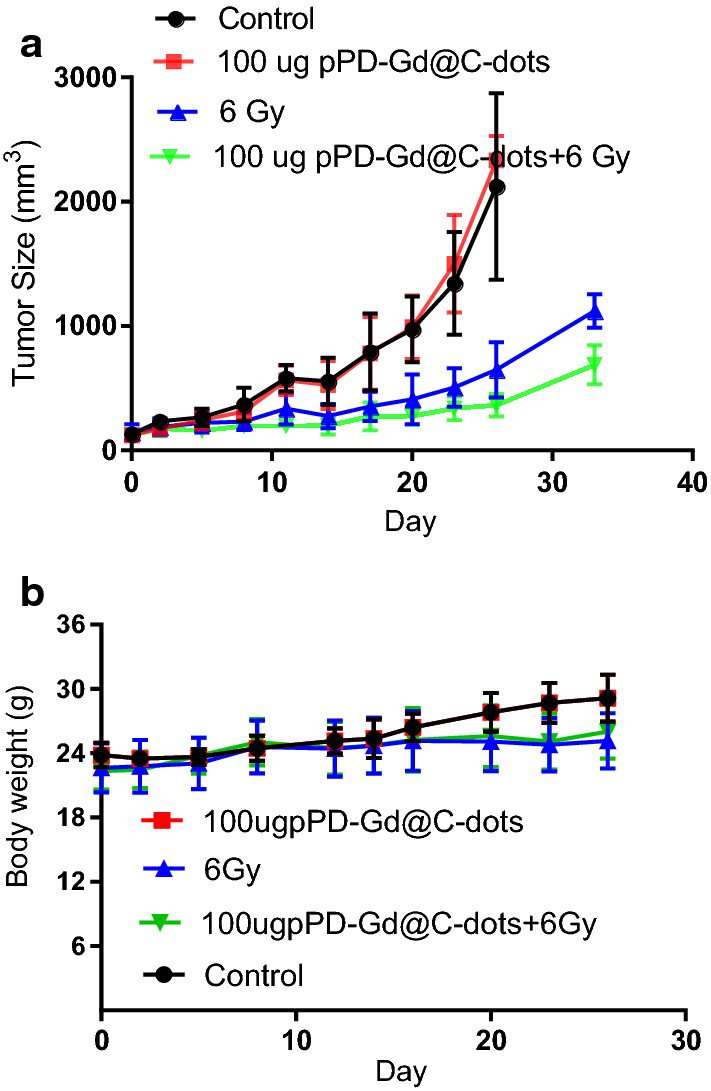
X-Ray radiotherapy studies on H1299 tumor models. Animals received one session of treatment on Day 0. **a** Tumor growth curves. Tumors were measured by monitoring tumor diameter changes at different time points. Compared to X-ray irradiation alone and pPD-Gd@C-dots injection only, significant tumor suppression was found with animals injected with pPD-Gd@C-dots and X-ray irradiation. **b** Body weight curves

Due to its high and persistent tumor retention, the following *in vivo* RT therapy studies were performed using pPD-Gd@C-dots in H1299 lung cancer model. In brief, the animals were randomly assigned to four groups: (1) No treatment, (2) 100 µg pPD-Gd@C-dots, (3) 6 Gy radiation, and (4) 100 µg pPD-Gd@C-dots + 6 Gy radiation. Our first set of experiment include single dose treatment and the results were shown in Fig. 8a. No treatment and 100 µg pPD-Gd@C-dots group showed fast tumor progression, and the tumor reached an end point within 28 days. In contrast, 6 Gy irradiation clearly demonstrated certain inhibitory effect on tumor growth, and the treatment efficacy was further improved once pPD-Gd@C-dots were used as a combination therapy. No significant weight drop was found in all treatment groups (Fig. 8b).

**Fig. 9:**
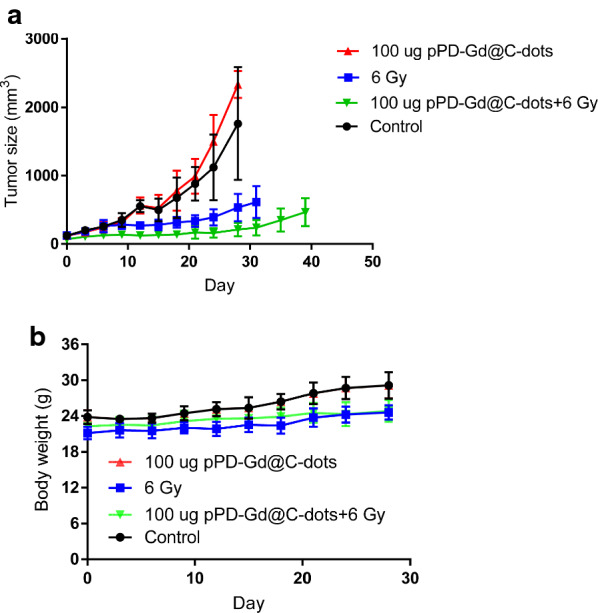
X-Ray radiotherapy studies on H1299 tumor models. Animals received two doses of treatment on Days 0 and Day 3. **a** Tumor growth curves. Tumors were measured by monitoring tumor dimension changes at different time points. Compared to X-ray irradiation alone and pPD-Gd@C-dots injection only, significant tumor suppression was found with animals treated with pPD-Gd@C-dots plus irradiation. **b** Body weight curves

According to the imaging study, pPD-Gd@Cdots have high tumor retention overtime. We then evaluated whether introducing a second 6 Gy irradiation (without re-injecting nanoparticle) could further improve treatment efficacy. As shown in Fig. 9, the tumor inhibition efficacy was further improved in the combination treatment group. No apparent body weight loss was observed. As shown in Figs. 8a and 9a, the tumor size increased significantly at late time points which may indicate the recurrence of the disease [38, 39]. In clinic, multi-session fractionated radiotherapy with a cumulative dose of ~ 60 Gy could be applied to lung cancer management. Due significant normal tissue toxicity, multi-session radiotherapy is not feasible for small animals. Whether the Cdots could achieve better therapeutic effect in clinic settings are still need to be investigated.

## Conclusion

Gd@C-dots have demonstrated the great potential to be used as radiation sensitizer due to their high stability, minimal metal leakage, and remarkable radiosensitizing effects. In this report, we demonstrated that PET offers a simple and straightforward way to study nanoparticle retention *in vivo*. The surface functional group could have significant impact on nanoparticle uptake and retention. The selected pPD-Gd@C-dots hold great potential as an effective radiosensitizer. We also like to point out that even though we did not observe significant toxicity of Gd@C-dots in the current and previous studies [40–42], potential long-term side effects of these carbon dots still need to be evaluated further in follow-up studies, especially at high doses. The obtained information will be critical to clinical translation of these formulations in the future.

## Supplementary Information

**Additional file 1**: **Figure S1**. Photos of pPD-Gd@C-dots (left) and CA-Gd@C-dots (right) in water. The solutions appeared dark red (pPD-Gd@C-dots) and brown (CA-Gd@C-dots), respectively. **Figure S2**. Cytotoxicity of pPD-Gd@C-dots and CA-Gd@C-dots, tested on H1299 cells using ATP bioluminescence assay. **Figure S3**. FT-IR spectra of Gd-CA@C-dots and pPD-Gd@C-dots, recorded on a Thermo Nicolet™ iS™ 10 FTIR Spectrometer. **Figure S4**. Hydrodynamic sizes of Gd-CA@C-dots and pPD-Gd@C-dots, measured on a Malvern Zetasizer Nano ZS system. The average diameters are 2.13 and 2.05, respectively.
