# Correction to: Image-guided selection of Gd@C-dots as sensitizers to improve radiotherapy of non-small cell lung cancer

**DOI:** 10.1186/s12951-021-01184-w

**Published:** 2022-01-03

**Authors:** Xiaofen Ma, Chaebin Lee, Tao Zhang, Jinghua Cai, Hui Wang, Fangchao Jiang, Zhanhong Wu, Jin Xie, Guihua Jiang, Zibo Li

**Affiliations:** 1 Department of Nuclear Medicine, Guangdong Second Provincial General Hospital, 466 Xingang Middle Road, Haizhu District, 510317 Guangzhou City, Guangdong Province People’s Republic of China; 2 Department of Radiology, Biomedical Research Imaging Center, and Lineberger Comprehensive Cancer Center, University of North Carolina at Chapel Hill, 125 Mason Farm Road, Chapel Hill, NC 27599 USA; 3 Department of Chemistry, University of Georgia, 140 Cedar Street, Athens, GA 30602 USA

## Correction to: J Nanobiotechnol (2021) 19:284 https://doi.org/10.1186/s12951-021-01018-9

Following publication of the original article [1] the authors have identified an error in Fig. 8 and 9. The correct version of figures are shown below.

**Fig. 8:**
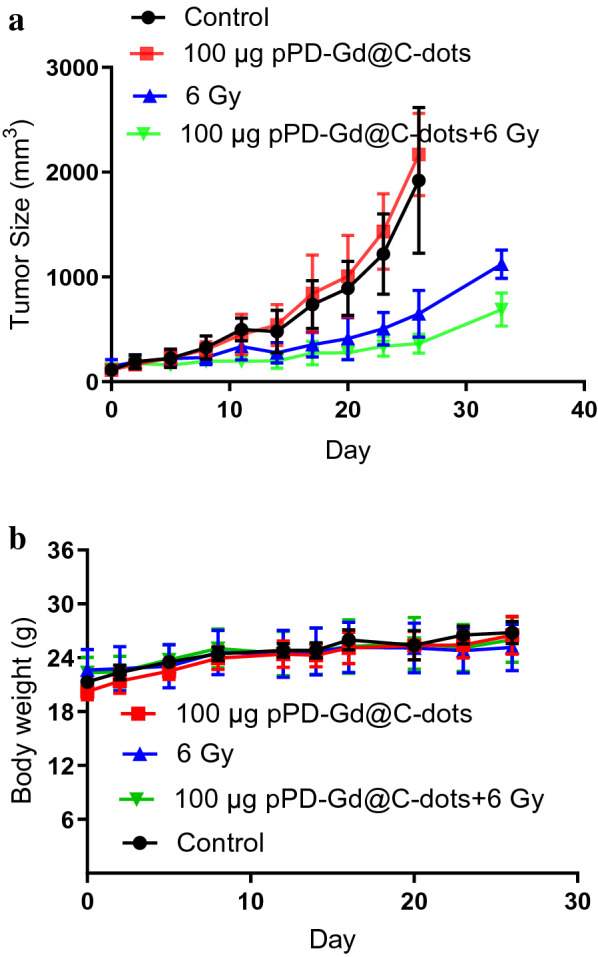
X-Ray radiotherapy studies on H1299 tumor models. Animals received one session of treatment on Day 0. **a** Tumor growth curves. Tumors were measured by monitoring tumor diameter changes at different time points. Compared to X-Ray irradiation alone and pPD-Gd@C-dots injection only, significant tumor suppression was found with animals injected with pPD-Gd@C-dots and X-ray irradiation. **b** Body weight curves

**Fig. 9:**
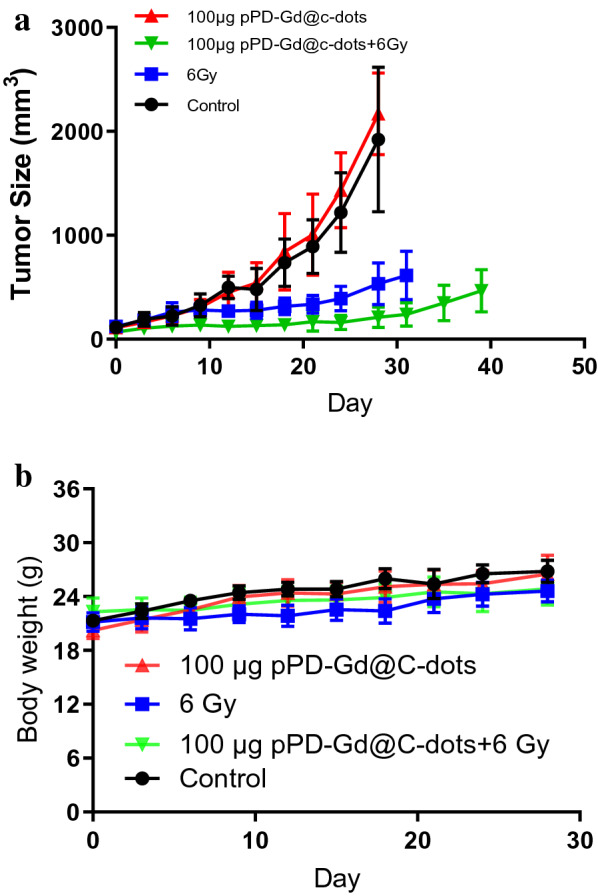
X-Ray radiotherapy studies on H1299 tumor models. Animals received two doses of treatment on Days 0 and Day 3. **a** Tumor growth curves. Tumors were measured by monitoring tumor dimension changes at different time points. Compared to X-Ray irradiation alone and pPD-Gd@C-dots injection only, significant tumor suppression was found with animals treated with pPD-Gd@C-dots plus irradiation. **b** Body weight curves

The authors apologise for this error.
