# Supplementary material for: Image-guided selection of Gd@C-dots as sensitizers to improve radiotherapy of non-small cell lung cancer
Source: J Nanobiotechnology. 2021 Sep 22;19:284. doi: 10.1186/s12951-021-01018-9 (PMC8456633; doi:10.1186/s12951-021-01018-9)
Supplement: Supplementary file 1 — Additional file 1: Figure S1. Photos of pPD-Gd@C-dots (left) and CA-Gd@C-dots (right) in water. The solutions appeared dark red (pPD-Gd@C-dots) and brown (CA-Gd@C-dots), respectively. Figure S2. Cytotoxicity of pPD-Gd@C-dots and CA-Gd@C-dots, tested on H1299 cells using ATP bioluminescence assay. Figure S3. FT-IR spectra of Gd-CA@C-dots and pPD-Gd@C-dots, recorded on a Thermo Nicolet™ iS™ 10 FTIR Spectrometer. Figure S4. Hydrodynamic sizes of Gd-CA@C-dots and pPD-Gd@C-dots, measured on a Malvern Zetasizer Nano ZS system. The average diameters are 2.13 and 2.05, respectively. [file 12951_2021_1018_MOESM1_ESM.docx]

Additional file 1


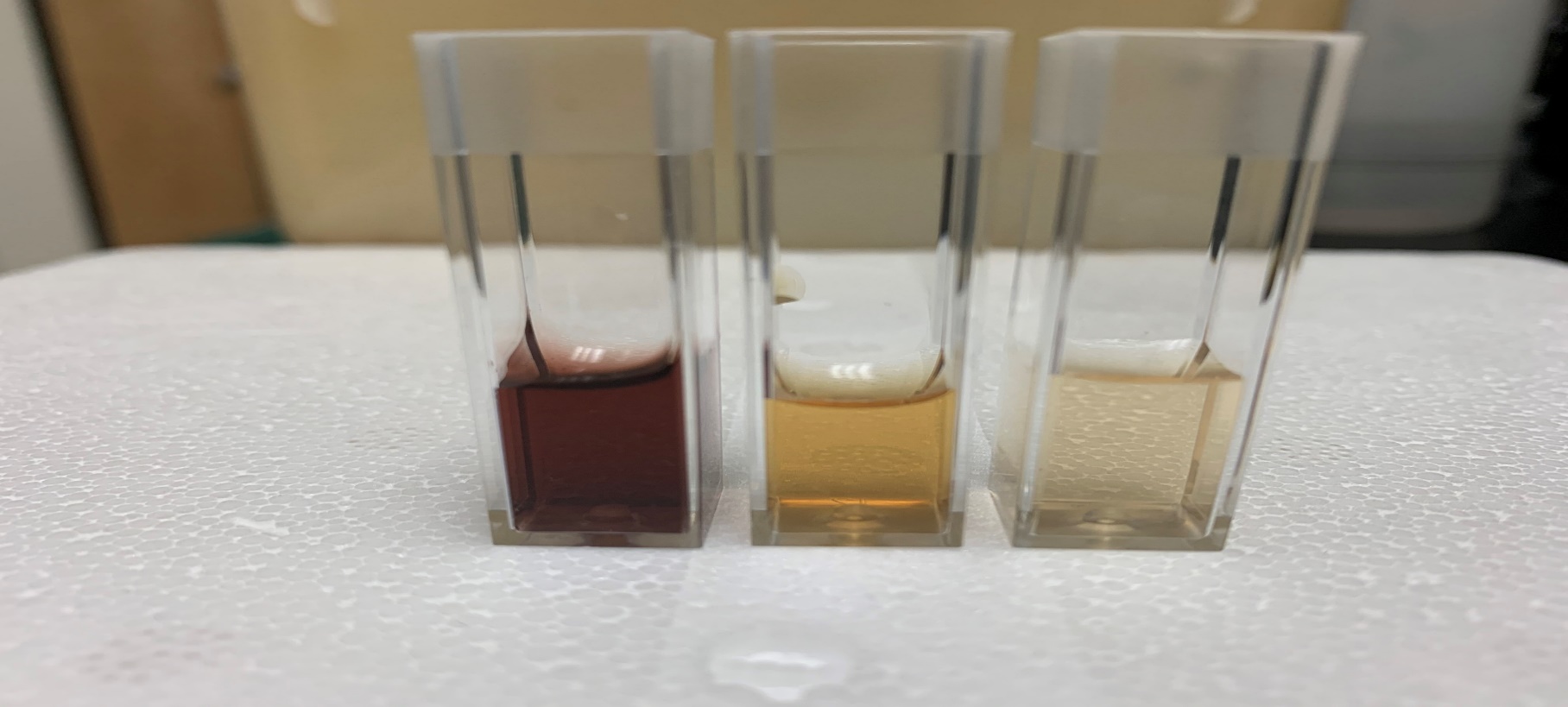


Figure S1. Photos of pPD-Gd@C-dots (left) and CA-Gd@C-dots (right) in water. The solutions appeared dark red (pPD-Gd@C-dots) and brown (CA-Gd@C-dots), respectively.

Figure S2. Cytotoxicity of pPD-Gd@C-dots and CA-Gd@C-dots, tested on H1299 cells using ATP bioluminescence assay.

Figure S3. FT-IR spectra of Gd-CA@C-dots and pPD-Gd@C-dots, recorded on a Thermo Nicolet™ iS™ 10 FTIR Spectrometer

Figure S4. Hydrodynamic sizes of Gd-CA@C-dots and pPD-Gd@C-dots, measured on a Malvern Zetasizer Nano ZS system. The average diameters are 2.13 and 2.05, respectively.
